# Biomechanical Effects of Various Bone-Implant Interfaces on the Stability of Orthodontic Miniscrews: A Finite Element Study

**DOI:** 10.1155/2017/7495606

**Published:** 2017-06-19

**Authors:** Fabing Tan, Chao Wang, Chongshi Yang, Yuanding Huang, Yubo Fan

**Affiliations:** ^1^College of Stomatology, Chongqing Medical University, Chongqing, China; ^2^Chongqing Key Laboratory of Oral Diseases and Biomedical Sciences, Chongqing, China; ^3^Chongqing Municipal Key Laboratory of Oral Biomedical Engineering of Higher Education, Chongqing, China

## Abstract

**Introduction:**

Osseointegration is required for prosthetic implant, but the various bone-implant interfaces of orthodontic miniscrews would be a great interest for the orthodontist. There is no clear consensus regarding the minimum amount of bone-implant osseointegration required for a stable miniscrew. The objective of this study was to investigate the influence of different bone-implant interfaces on the miniscrew and its surrounding tissue.

**Methods:**

Using finite element analysis, an advanced approach representing the bone-implant interface is adopted herein, and different degrees of bone-implant osseointegration were implemented in the FE models. A total of 26 different FE analyses were performed. The stress/strain patterns were calculated and compared, and the displacement of miniscrews was also evaluated.

**Results:**

The stress/strain distributions are changing with the various bone-implant interfaces. In the scenario of 0% osseointegration, a rather homogeneous distribution was predicted. After 15% osseointegration, the stress/strains were gradually concentrated on the cortical bone region. The miniscrew experienced the largest displacement under the no osseointegra condition. The maximum displacement decreases sharply from 0% to 3% and tends to become stable.

**Conclusion:**

From a biomechanical perspective, it can be suggested that orthodontic loading could be applied on miniscrews after about 15% osseointegration without any loss of stability.

## 1. Introduction

Miniscrew has been extensively applied in orthodontic treatment as a temporary anchorage device because of its ease of placement, low cost, minimal anatomic limitations, and enhanced patient comfort. The existing evidence suggests a success rate of more than 80% for miniscrews [1]. Likewise, Albogha and Takahashi have stated a success rate ranging from 77.7% to 93.43% in their study [2]. However, the failure of miniscrew may have dramatic consequences and remain difficult to be anticipated by orthodontists [3]. Since the failure of miniscrew necessitates additional surgical interventions and prolonged treatment time, investigating the mechanical stability of miniscrew becomes imperative.

The biomechanical properties of bone to implant interface are the key determinants for miniscrew stability. Initially, when the miniscrew is placed into bone, the retention of the implant is provided by mechanical locking. Later, with the progression of bone formation around the implant, bioactive retention can be achieved via physicochemical bonding. It is clinically evident that full osseointegration is a prerequisite for successful prosthetic (or dental) implants [4, 5]. Nevertheless, some fibrous tissue formation at the bone-implant interface would be acceptable because orthodontic loading has to be applied as early as possible and also the miniscrew at the end of treatment must be easily removable [3]. That is to say, partial bone-implant osseointegration of the miniscrew might be permitted for orthodontic treatment. Therefore, the effect of the different degrees of bone-implant osseointegration on the stability will be of great interest from the orthodontist's point of view.

The objective of this study was to investigate the influence of the different implant-bone interface conditions on the biomechanics of an orthodontic miniscrew and its surrounding tissue with the use of finite element analysis (FEA). FEA is particularly suitable for a biological structure analysis as it allows great flexibility in dealing with geometric complex domains composed of multiple materials [2, 6–8]. In the present study, an advanced approach representing the bone implant interfaces was adopted wherein [9] different percentages of bone-implant osseointegration were implemented in the FE models and the biomechanical behavior of miniscrew and the supporting tissue with the various bone-implant interfaces was predicted and compared.

## 2. Materials and Methods

**Figure 1:**
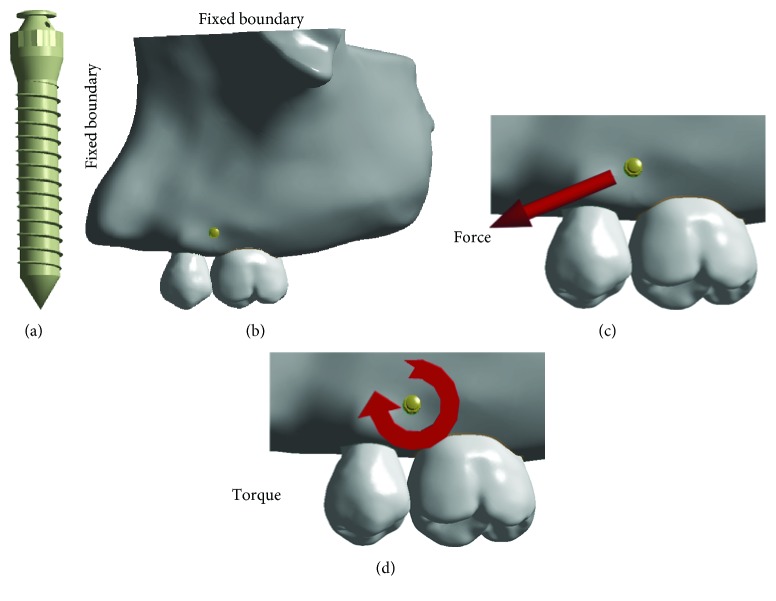
(a) Microimplant. (b) Geometry models with fixed boundary conditions (buccal side). (c) The traction force and (d) revolving torque employed during orthodontic loading.

The geometry of the partial maxilla, including both premolar and molar, was obtained from the dental hospital, and computed tomography images captured at 0.5 mm intervals were disposed with Mimics software (Materialise NV, Leuven, Belgium) and Geomagic Studio software (Geomagic Company, NC, USA). Maxillary trabecular bone was modeled as a solid structure in the cortical bone with an average thickness of 2 mm based on the CT images. Likewise, the periodontal ligament (PDL) was modeled based on the external geometry of teeth roots with a thickness of 0.20 mm. The implant was structured as a threaded endosseous miniscrew (8 mm length, 1.3 mm diameter, 0.1 mm thread ridge, 60 degrees screw top angle, and 0.5 mm thread pitch) by using a commercial CAD software SolidWorks (SolidWorks Corp., Dassault Systemes, Concord, MA, USA). The miniscrew was inserted into maxillary bone between the premolar and molar at a distance of 3 mm from the alveolar crest, as shown in Figure 1.

**Figure 2:**
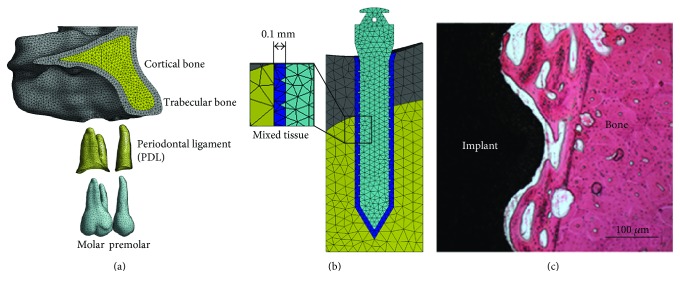
(a) Finite element models of cortical bone, trabecular bone, periodontal ligament (PDL), and premolar and molar (palatal side). (b) The 0.1 mm (100 *μ*m) thick mixed tissue around microimplant. (c) The histological image showing the bone-implant interface (courtesy of Professor Shicheng Wei).

The entire model was imported to the finite element package ANSYS Workbench (Swanson Analysis System Co., Houston, TX, USA). The finite element model was meshed using 10-node solid tetrahedral elements (Figure 2(a)). Following a convergence test [7], 0.5 mm was determined to be the appropriate element mesh size for bone and tooth, and even a miniature size (0.2 mm) was selected to accommodate the small feature in the model (e.g., PDL and miniscrew). The detailed element assignment is listed in Table 1. The contacts among the tooth, the related bones, tissue, and ligaments are defined in Table 2.

**Figure 3:**
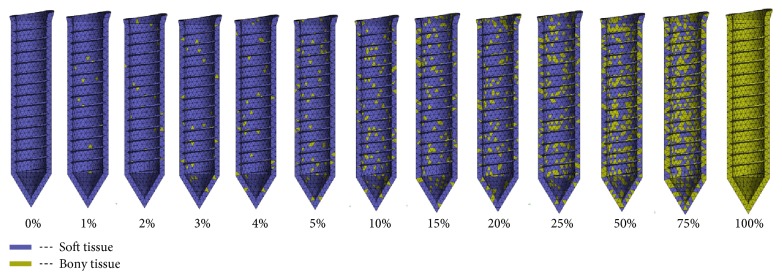
Different degrees of the bone-implant osseointegration interfaces implemented in the FE models, varying from 0% to 100%.

For the realistic presentation, different amounts of bone-implant osseointegration were implemented, ranging from 0% to 100% (Figure 3). In the existing studies, it was found that there should be a small gap between the implant and peri-implant bone [9, 10]. To evaluate the effect of different bone implant interfaces, a simulation method has already been developed by Lian et al. [9], which was used in the present study. Hence, based on the histological image (Figure 2(c)) [11], it can be suggested that 0.1 mm (100 *μ*m) thick mixed tissue exists around the miniscrew, constituting a blend of bony tissue and soft tissue to simulate varying bone-implant contact (Figure 2(b)). An ad hoc APDL (ANSYS Parametric Design Language) routine was developed to set the different bone-implant osseointegration. As shown in Figure 3, a certain percentage of mixed tissue elements were selected randomly and assigned the properties of bony tissue. The remaining elements within the mixed tissue were designated as soft tissue. In this study, a total of 13 different percentages of bone-implant osseointegration were considered (0%, 1%, 2%, 3%, 4%, 5%, 10%, 15%, 20%, 25%, 50%, 75%, and 100%).

Mesial and superior maxillary bone surfaces were fixed in all directions as the boundary conditions (Figure 1(b)). To consider the loading effect of different clinical applications [9, 12], two different kinds of orthodontic load (traction force and revolving torque) were applied at the head of the screw (Figures 1(c) and 1(d)). The direction of the traction force applied was 30 degrees declination to the occlusal plane (Figure 1(c)), and the revolving torque was applied in the clockwise direction (Figure 1(d)).

Fully anisotropic elastic components were used for both cortical and trabecular bones [13, 14], as listed in Table 3. A nonlinear elastic stress-strain behavior of PDL was employed and inputted into FE models, following the approach proposed by Toms et al. [14]. Miniscrew and dentin were considered homogeneous, isotropic, and linearly elastic (Table 4).

## 3. Results

In the present study, a total of 26 analyses were performed, including the 13 different degrees (from 0% to 100%) of bone-implant osseointegration models with two different kinds of orthodontic load applications (traction force and revolving torque).

**Figure 4:**
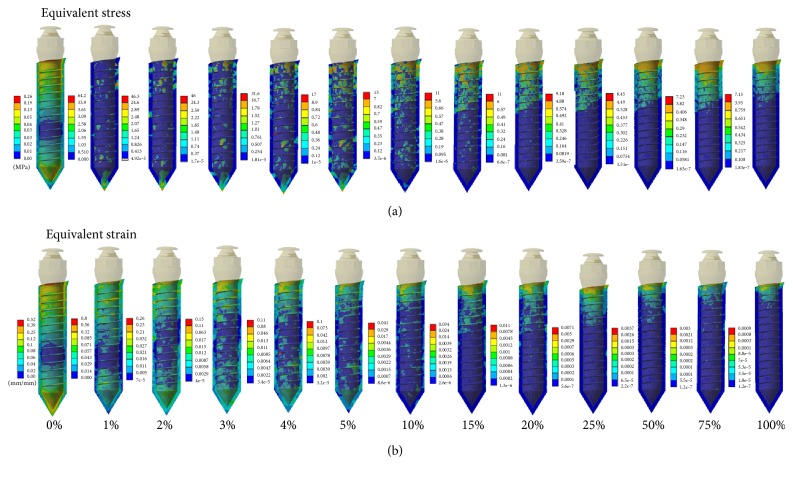
Progressive alteration of (a) equivalent stress and (b) strain distributions in peri-implant tissue under the application of traction force.

**Figure 5:**
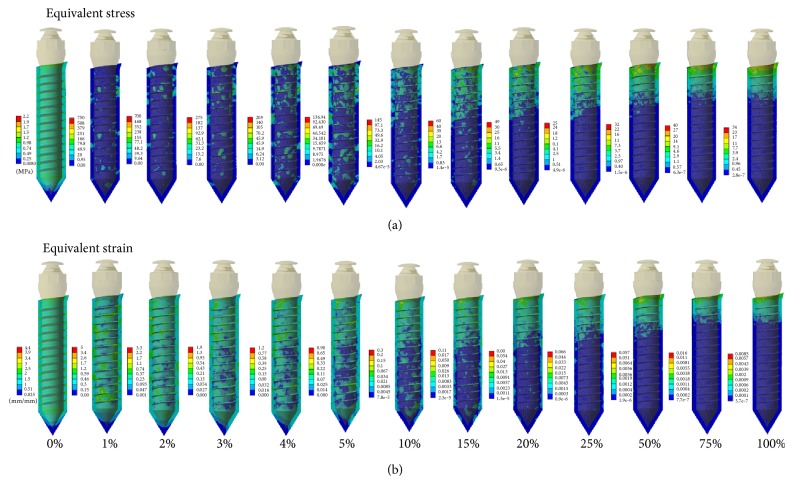
Progressive alteration of (a) equivalent stress and (b) strain distributions in peri-implant tissue under the application of revolving torque.

The FE simulated results for the force and torque load in the peri-implant tissue (mixed tissue region) are shown in Figures 4 and 5, respectively. For the ease of observation, equivalent stresses/strains in the cross section of the FE models are displayed. As shown in the figures, the stress and strain distributions in the mixed tissue are changing with the various bone-implant interfaces. Initially, in the scenario of 0% osseointegration, a rather homogeneous equivalent stress/strain distribution was predicted. And then, remarkable stress/strain concentrations could be seen in the peri-implant tissue with the 1% osseointegration interface. After the 15% osseointegration, the stress and strains were gradually concentrated on the cortical bone region rather than in the trabecular bone region. It is worth noting that, whatever kind of the orthodontic loads are subjected, there is a significant change in the first 7 degrees (0%~10%), but the variation range reduced obviously after the 15% osseointegration.

**Figure 6:**
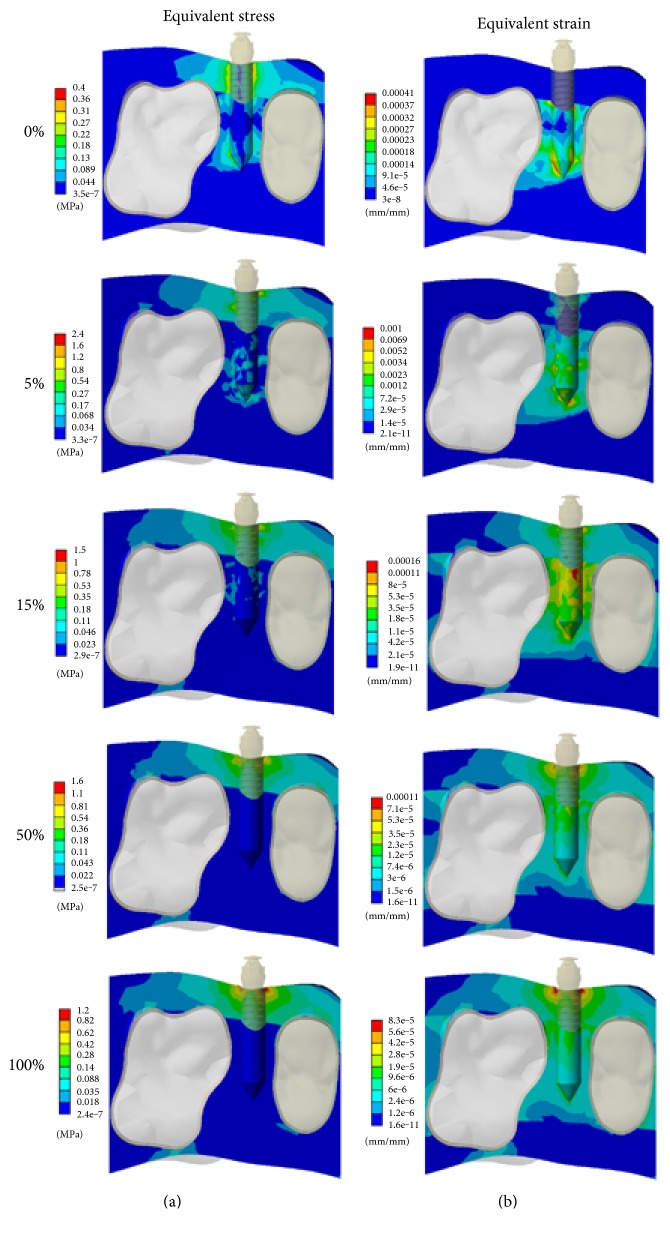
Progressive alteration of (a) equivalent stress and (b) strain distributions in the surrounding bone under the application of traction force.

**Figure 7:**
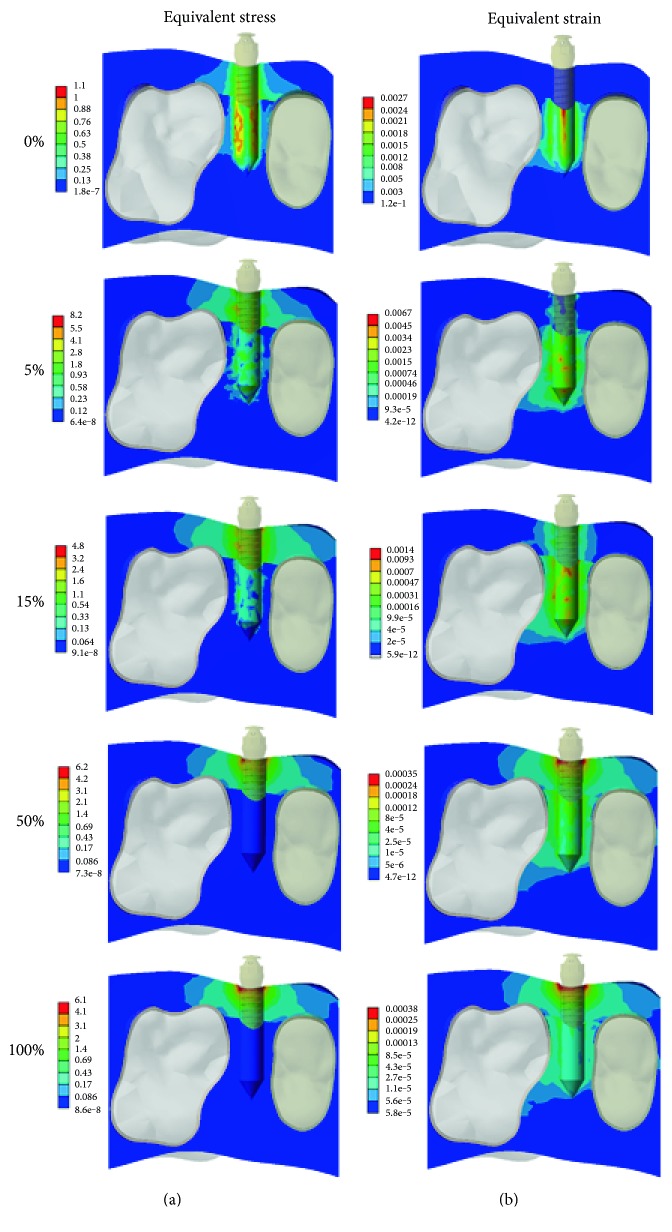
Progressive alteration of (a) equivalent stress and (b) strain distributions in the surrounding bone under the application of revolving torque.

Figures 6 and 7 show the equivalent stress and strain on the surrounding bone under the application of traction force and revolving torque, respectively. As evident from Figure 6, under the application of traction force, the stress induced in the cortical bone was much higher as compared to that in the trabecular bone. With the change in bone-implant interfaces, the stress distribution gradually concentrated on the bone around the neck of miniscrew. The strain distribution also showed a trend similar to the stress. However, in the initial phase (0%~15%), the maximum strain was located in the trabecular bone rather than the cortical bone. Furthermore, the equivalent stress and strain distributions with revolving torque application are shown in Figure 7. The changing trend of equivalent stress and strain is much similar to that of traction force application. At the beginning (0% osseointegration), the stress was longitudinally distributed along the whole miniscrew. With the integration of the bone and implant, the stress was highly concentrated in the neck of the miniscrew. Similarly, the strain distribution was concentrated on the trabecular bone initially, which later on shifted towards the neck of the miniscrew with the change in bone-implant osseointegration percentages.

**Figure 8:**
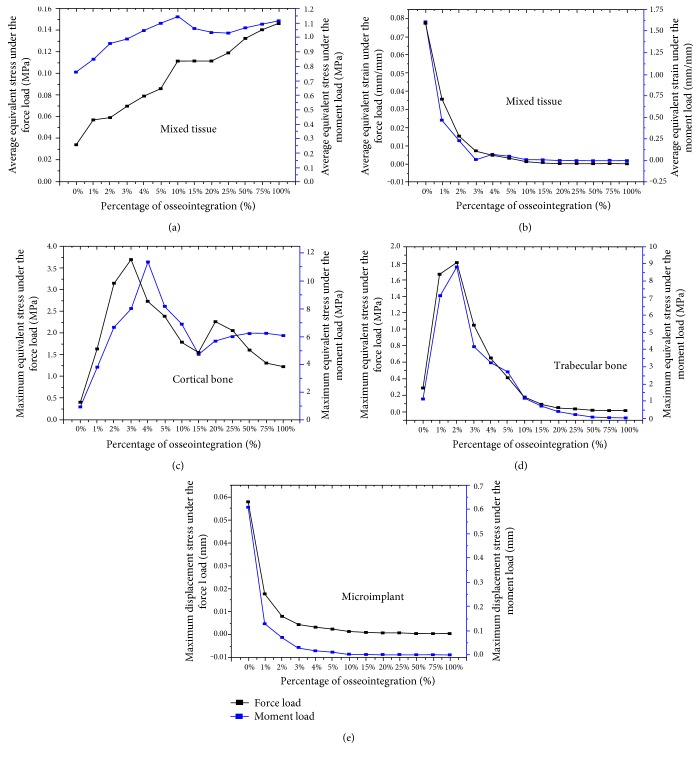
Change in biomechanical characteristics of microimplant and supporting oral tissues with the different bone-implant osseointegration interfaces. (a) The average equivalent stress in the peri-implant mixed tissue. (b) The average equivalent strain in the peri-implant tissue. (c) The maximum equivalent stress in cortical bone. (d) The maximum equivalent stress in trabecular bone. (e) The maximum displacement of microimplant.

Figure 8 illustrates quantitatively the relationship between the degree of bone-implant osseointegration and biomechanical characteristics of bone-implant complex. Figures 8(a) and 8(b) represent the change in average equivalent stress and strain in the mixed (peri-implant) tissue, respectively. It is evident from Figure 8(a) that the stress increases progressively before the 10% osseointegration, followed by a slight decrease, and then increases again. As shown in Figure 8(b), the strain decreases significantly at the beginning, and then tends towards stability until the 100% osseointegration is achieved. From Figures 8(c)and 8(d) , it can be seen that the equivalent stress changes with the increase in the osseointegration degrees in the cortical bone and trabecular bone region, respectively. Besides, initially the stress increases sharply, and then drops down followed by a gradual increase again after the 15% osseointegration. As shown in Figure 8(d), it can be seen that the graph exhibits similar patterns to those presented in Figure 8(c), but a turning point is not observed at the 15% osseointegration in the trabecular bone. Relative to the displacement (Figure 8(e)), the miniscrew experienced the largest displacement under the condition of no osseointegration (0%). The maximum displacement decreases sharply from 0% to 3% and tends to become stable after completion of approximately 3~4% osseointegration.

## 4. Discussion

In this study, finite element models were generated to investigate the effect of different implant-bone interface conditions on the mechanical stability of miniscrew. From 0% to 100%, 13 different degrees of bone-implant interfaces were simulated. The stress/strain patterns generated by the miniscrews at the surrounding tissue were calculated and compared, and the displacement of miniscrew was also evaluated.

In dental biomechanics, almost all the FE models generated currently simulated different bone to implant interfaces by employing frictional contact analysis [2]. In a typical FE model built by Yang and Xiang [15], three different contact types were used to represent the integration quality at the implant-bone interface. The “bonded” type simulates a full osseointegration; the “no separation” type indicates an imperfect osseointegration, and the “frictionless” contact implies no osseointegration. As a progressive technology of simulating partial contact, a random algorithm was developed by Gracco et al. to set a part of the nodes localized at the bone/implant interface as the tie constraint, and the remaining part of the interface was set as frictional contact [16]. However, although contact analyses with different frictional coefficients can be used to assess the biomechanical effects of many different implant-bone complex, the specific frictional coefficients is still difficult to determine by an existing biomechanical testing [17]. In order to overcome the limitations of the existing methodologies, an alternative method was proposed by Lian et al. [9]. In this method, an assumption was made that a small part of tissue surrounding the implant was constituted as a mix of hard and soft tissue. According to the observation of previous histological studies [18, 19], this assumption has been proved to be reasonable. Therefore, considering the progressive change of the surrounding tissue around the miniscrew, this alternative method was advanced from a 2D simulation to 3D, to reproduce the different bone-implant interfaces in our FE models.

Till date, the minimum level of bone-implant osseointegration for clinical success in orthodontics has not been clearly described. From the biomechanical viewpoint, the minimum amount of bone-implant osseointegration required can be inferred from our analytical results. For the equivalent stress and strain (Figures 4–7), the implant-bone interface conditions significantly affected the stress/strain distributions on the surrounding tissue when the osseointegration was less than 15%. Further analysis (15%~100%) reveals that, even though the stress/strain concentration appears around the implant neck region, the overall changes in the stress/strain distributions from 15% to 100% osseointegration can be neglected. Besides, according to the progressive biomechanical characteristics of bone-implant complex (Figure 8), the minimum amount of bone-implant osseointegration may vary between 2% and 10%. Some of the previous findings are also in support of our results. Deguchi et al. implied that implants with as little as 5% bone osseointegration at the bone-implant interface can successfully withstand orthodontic force [20], and also the study by Woods suggested that 2.2 percent BIC may be sufficient for light force [21]. However, during the low degrees of bone-implant osseointegration (0~15%), our results show that the presence of connective tissue (soft tissue) at the implant-bone interface might result in an increase of stress/strain magnitudes in the trabecular bone as compared with full osseointegration conditions. Because of the occurrence of fibrous tissue, miniscrew cannot be tightly held by alveolar bone, leading to miniscrew instability. The surrounding trabecular bone might be damaged due to the changing mechanical environment induced by the miniscrew, and excessive implant displacement may cause loosening, dislocation, or even loss of the implant. Therefore, it can be inferred that orthodontic loading can be applied over the miniscrew after completion of 15% osseointegration without a compromise of stability. That is to say, less than 15% osseointegration might be a risk factor in terms of implant stability, and hence should be avoided.

Now, a critical question arises, that is, what should be the latency period to achieve the minimum percentage of bone-implant osseointegration during an orthodontic treatment? In reference to previous animal/ histological studies, several investigations have been done on the healing time of orthodontic miniscrew. However, no study has been conducted specifically to solve this problem. Even more, the existing results are inconclusive about the proper timing of orthodontic force application. A histological study done by Ramazanzadeh et al. concluded that healing time has no significant effect on miniscrew stability, but only a comparison of bone-implant contact between 4 weeks and 8 weeks was made in his study [22]. In an another study by Oltramari-Navarro et al., similar histomorphometric results were observed for the immediate and the delayed orthodontic loads groups, but it is important to note that the immediate group presented higher failure rate (50%) than the delayed group [18]. Likewise, the results of an animal study by Zhao et al. indicated that early loading may decrease the osseointegration of miniscrews, and the same investigators suggested that a 4-week healing time is recommended before orthodontic loading [23]. Deguchi et al. also concluded that a minimal healing period of 3 weeks is required for orthodontic loading [20]. Above all, the existing animal experiments presented some useful conclusion; however, their results remain limited when it comes to understanding the various conditions of bone-implant interfaces playing a role in miniscrew stability.

The research limitations and suggestions for future research should be pointed out. Firstly, additional animal research is required to answer the above-mentioned question. If the exact time for achieving 15% osseointegration of miniscrew could be confirmed, the appropriate time of miniscrew loading can be effectively ensured for orthodontists. Secondly, the bone remodeling process was not considered in the simulation. In fact, the bone remodeling occurs around the implant during the healing period. So the progressive process of bone remodeling should be included in further simulation to investigate mechanical stability of orthodontic miniscrew. Finally, the material nonlinear properties of the mixed tissue (hard and soft tissue) should be considered in the FE analysis. Because large deformation can be observed during this simulation, the incorporation of nonlinear properties can provide more accurate and reliable results.

## 5. Conclusions

Within the limitation of this study, it can be suggested that the orthodontic force can be applied at the miniscrew after completion of approximately 15% osseointegration which is the more beneficial for the mechanical stability of the miniscrew. Under this condition, the miniscrew can be tightly held in place by the surrounding tissue and employed as orthodontic anchorage without compromising implant stability. For clinical application of the results simulated in our study, a specifically designed study is required to confirm the appropriate time of orthodontic loading in the future.

## Figures and Tables

**Table 1:** The number of nodes and elements of FE model.

	Nodes	Elements
Cortical bone	150,450	94,395
Trabecular bone	161,301	107,895
Microimplant	7563	4000
PDL	28,913	14,332
Teeth	16,793	9384
Mixed tissue	56,068	29,579
Total	421,088	259,585

**Table 2:** Contact types set in the FE models.

Contact bodies		Contact type
Tooth	Tooth	Frictionless
Tooth	PDL	Bonded
PDL	Bone	Bonded
Microimplant	Mixed tissue	Bonded
Mixed issue	Bone	Bonded

**Table 3:** Anisotropy elastic coefficients for cortical and trabecular bone^a^.

	E1	E2	E3	G12	G13	G23	ν12	ν13	ν23
Cortical bone^b^	12.5	17.9	26.6	4.5	5.3	7.1	0.18	0.31	0.28
Trabecular bone^c^	0.21	1.148	1.148	0.068	0.068	0.434	0.055	0.055	0.322

^a^ ^b^ ^c^E_*i*_ represents Young's modulus (GPa); G_*ij*_ represents shear modulus (GPa); ν_*ij*_ represents Poisson's ratio; the 1-direction is radial, the 2-direction is tangential (circumferential), and the 3-direction is axial (longitudinal); the 1-direction is inferosuperior (the axis of transverse isotropy symmetry with the smallest of Young's modulus value), the 2-direction is mediolateral, and the 3-direction is anteroposterior.

**Table 4:** Material elastic modulus parameter employed in the FE models.

	Elastic modulus (GPa)	Poisson's ratio	References
Tooth	22.0	0.31	Holberg et al., 2013
Microimplant	113.4	0.342	Alrbata et al., 2014
Bony tissue	2.4	0.3	Lian et al., 2010
Soft tissue	0.07	0.3	Lian et al., 2010
